# Editing the genome of hiPSC with CRISPR/Cas9: disease models

**DOI:** 10.1007/s00335-017-9684-9

**Published:** 2017-03-16

**Authors:** Andrew R. Bassett

**Affiliations:** Wellcome Trust Sanger Institute, Wellcome Genome Campus, Hinxton, Cambridge, CB10 1SA UK

## Abstract

The advent of human-induced pluripotent stem cell (hiPSC) technology has provided a unique opportunity to establish cellular models of disease from individual patients, and to study the effects of the underlying genetic aberrations upon multiple different cell types, many of which would not normally be accessible. Combining this with recent advances in genome editing techniques such as the clustered regularly interspaced short palindromic repeat (CRISPR) system has provided an ability to repair putative causative alleles in patient lines, or introduce disease alleles into a healthy “WT” cell line. This has enabled analysis of isogenic cell pairs that differ in a single genetic change, which allows a thorough assessment of the molecular and cellular phenotypes that result from this abnormality. Importantly, this establishes the true causative lesion, which is often impossible to ascertain from human genetic studies alone. These isogenic cell lines can be used not only to understand the cellular consequences of disease mutations, but also to perform high throughput genetic and pharmacological screens to both understand the underlying pathological mechanisms and to develop novel therapeutic agents to prevent or treat such diseases. In the future, optimising and developing such genetic manipulation technologies may facilitate the provision of cellular or molecular gene therapies, to intervene and ultimately cure many debilitating genetic disorders.

## Introduction

### Current genetic models of disease

Thousands of human diseases are known to have a genetic component, although the penetrance of this effect and the contribution of environmental influences are highly variable. Recent advances in genotyping and DNA sequencing have facilitated the studies of familial inheritance, de novo mutations (Deciphering Developmental Disorders 2015; Wright et al. 2015) and numerous genome-wide association studies (GWAS) (Visscher et al. 2012), which have begun to identify the genetic loci underlying many of these diseases. However, despite such advances in human genetic analysis, unravelling the causative lesions, understanding the underlying molecular and cellular mechanisms and developing ways to prevent or treat such diseases still require experimental models (Nishizaki and Boyle 2016).

The evolutionary conservation of mammalian genomes, especially in protein coding sequence, has enabled the use of many animal models such as mice, rats and non-human primates for studying the effects of genetic lesions upon molecular, cellular, physiological and behavioural phenotypes. This has led to many important insights into disease biology, and their importance in such studies is undeniable. Despite such conservation of function, given the last common ancestor of human and mouse was around 100 million years ago (Mouse Genome Sequencing Consortium et al. 2002), it is unsurprising that there are also differences between these organisms. Around 20% of genes in humans lack an identifiable one-to-one orthologue in mouse (Mouse Genome Sequencing Consortium et al. 2002), and the number of paralogs within an organism is often different, many of which have diverged to provide subtly different functions (Gabaldon and Koonin 2013). Equally, even apparently orthologous genes can play different roles, such as in the case of TDP1, which shows a different subcellular localisation in humans and mice, and mutations in which are linked to the SCAN1 disorder in humans, but lack a clear phenotype in the mouse (Gharib and Robinson-Rechavi 2011). Additionally, there will clearly always be certain differences inherent to a particular species due to their evolutionary adaptation, for instance in cardiac or brain function between human and mouse, making it impossible to study some human-specific phenotypes in animal models.

One of the surprises of the human genome project (Lander et al. 2001) was that only a relatively small proportion of the genome is protein coding (current estimates are around 1.2%) (Pruitt et al. 2009). The remainder of the sequence contains many repetitive sequences and transposon remnants, although a further 3–10% of the human genome displays evidence of evolutionary conservation, implying its functionality (Lunter et al. 2006). There is clearly a role for at least a proportion of this non-coding sequence in regulation of gene expression. In fact, more than 95% of disease-associated single nucleotide polymorphisms (SNPs) lie within the non-coding genome (Maurano et al. 2012). Importantly, such SNPs may be functionally relevant, since they are enriched within enhancer regions (marked by DNAse hypersensitivity) specific for the disease-associated tissue (Maurano et al. 2012), and are often associated with changes in neighbouring gene expression (Degner et al. 2012). It is also beginning to become apparent that such non-coding changes can result in phenotypic effects, and be causative in certain diseases (Soldner et al. 2016). In the context of disease modelling, such sequences are much more poorly conserved between organisms than protein coding sequences, often making it impossible to identify the orthologous region in other species. In this situation, developing a human model of disease becomes even more relevant.

Primary cell cultures from human patients are an invaluable resource to study the molecular and cellular effects of particular mutations, but there are many limitations to this strategy, not least in the inaccessibility of certain tissues, for instance the brain. Even if the tissue is accessible, such cells are often challenging to culture, and cannot be maintained for extended periods of time, making genetic engineering difficult. Equally, many primary cultures consist of heterogeneous cell populations that are not necessarily consistent between samples, often complicating analysis. Although many immortalised cell lines also exist, these necessarily contain genetic aberrations that enable their continued culture, and therefore do not represent a highly physiological model of disease.

The generation of human embryonic stem cell (hESC) lines (Shamblott et al. 1998; Thomson et al. 1998) opened up the exciting possibility of using these pluripotent stem cells to study the function of differentiated derivative cell types. However, due to the technical and ethical difficulties, it is not feasible to produce a large number of such lines or derive them from patients with diseases, limiting their use to studies of normal cellular function, or to introduction of known engineered genetic changes.

## Induced pluripotent stem cells (iPSCs) for disease modelling

### iPSC technology

The advent of induced pluripotent stem cell (iPSC) technology (Takahashi et al. 2007; Takahashi and Yamanaka 2006) has revolutionised many fields, notably those of disease modelling and cellular therapeutics due to our ability to generate such pluripotent stem cells from essentially any human, including those with disease (Avior et al. 2016). Somatic cells can be reprogrammed to a pluripotent stem cell state similar to that present in very early embryogenesis through transient expression of four transcription factors (Oct4, Sox2, Klf4 and c-Myc) (Takahashi et al. 2007; Takahashi and Yamanaka 2006). Importantly, such cells are diploid and karyotypically normal, can self-renew for many cell divisions and can be differentiated into a broad range of different cell types. These characteristics lend themselves to the study of development and cellular function both in normal and disease states, and also allow large numbers of cells to be produced for high throughput genetic and drug screening as well as cell therapy. This has led to the inception of several large-scale initiatives for deriving iPSCs from thousands of normal and diseased patients (California Institute for Regenerative Medicine (CIRM), Stem Cells for Biological Assays of Novel Drugs and Predictive Toxicology (StemBANCC) (Morrison et al. 2015) and the Human-induced Pluripotent Stem Cell initiative (HiPSCi) (Streeter et al. 2016)). Cell lines have been thoroughly characterised by for example DNA sequencing, SNP genotyping, RNA sequencing and DNA methylation analysis (Soares et al. 2014) and can be accessed through cell banks across the world (such as the European Collection of Cell Cultures (ECACC), the European Bank for induced pluripotent Stem Cells (EBiSC) and the Coriell biorepository). These cell lines have been derived from individuals with a variety of monogenic and polygenic disorders, and provide an invaluable resource for studying genetic contributions to human disease. They can be used to create personalised models of disease, and understand the molecular and cellular phenotypes underlying their pathogenesis. Interestingly, since cells are reprogrammed to a very early stage of development, they can be used to monitor both developmental or differentiation defects as well as the temporal sequence of events in the early stages of disease progression.

### Considerations for iPSC disease models

When considering use of iPSCs as a disease model, there are many important considerations; whether the disease is monogenic or polygenic, the penetrance of the mutation, the age of onset, whether differentiation into an appropriate cell type is possible, and if there is an appropriate phenotypic readout at a molecular or cellular level.

Whilst the majority of genetic diseases are due to a small contribution from a large number of genes, such polygenic disorders are inherently more difficult to study than monogenic diseases, since typically both the penetrance and severity of the phenotype due to any single mutation are lower (Wheeler et al. 2016). This is true of any disease model, and our ability to obtain iPSCs from patients with and without a disease makes analysis of polygenic disorders such as autism (DeRosa et al. 2012) or schizophrenia (Brennand et al. 2011) more feasible. However, further genetic manipulations to prove the causal alleles (see below) become more challenging due to the larger number of genes involved, smaller phenotypic effects and the potential for epistasis between different alleles. As with most current models of such diseases, it is often simpler to study the effect of a familial form with higher penetrance and severity, to identify phenotypes that can then be recapitulated in other forms of the disease.

Equally, it is critical with any iPSC disease model to pinpoint a cell type in which the disease manifests, to be able to differentiate effectively into these cells, and to identify a molecular or cellular phenotypic readout of the disease state. Differentiation protocols are now available to efficiently generate a large variety of lineages, and many others are being developed using cocktails of small molecule inhibitors or transcription factor overexpression (Cohen and Melton 2011; Mertens et al. 2016; Murry and Keller 2008). Although such protocols often result in a mixed population, purification of the desired cells by for example fluorescence-activated cell sorting (FACS) using an appropriate marker or reporter gene can be used to enrich for the population of interest (Horikiri et al. 2017; Wu et al. 2016a, b). Perhaps more critical to the success of any cellular disease model is the identification of a molecular or cellular phenotype that correlates with the disease state. In many cases, this can be identified through global gene expression profiling of patient and control samples (at the RNA or protein level), and identification of a profile of gene expression changes that correlate with disease. Alternatively, other cellular phenotypes can be employed such as functional readouts of cell activity (e.g. electrophysiological measurements of neurons, activity of cardiac muscle or response of macrophages to pathogen stimulation), more generic cellular features such as cell shape, subcellular localisation of particular marker genes, endocytic trafficking, or cellular responses to their environment (e.g. secretion or response to signals, sensitivity to drugs or other cellular stresses). In some cases, such as mutations in SCN5A that are linked to cardiac arrhythmia and long QT syndrome, these phenotypes are predictable and directly related to the disease (Davis et al. 2012). However, in other diseases such as autism spectrum disorders (ASD) (DeRosa et al. 2012) or schizophrenia (Brennand et al. 2011) which are classified by complex behavioural phenotypes, how well these correlate to any underlying molecular or cellular changes, and to what extent these are causative in the disease are still largely unexplored.

Another important consideration with the use of iPSCs in disease modelling is that these cells and their differentiated derivatives often resemble those of foetal origin (Hrvatin et al. 2014), and therefore the age of onset of any disease becomes relevant. Indeed, iPSC-derived neurons initially differentiate into an immature state and can require months in culture before they become electrophysiologically active. This complicates analysis of diseases such as neurodegeneration which only show effects late in life. Several strategies exist to circumvent this issue, at least to some extent. Often, rare, early-onset, familial mutations are associated with many normally polygenic late-onset diseases, and these can be useful models to study phenotypes associated with such diseases in general. One example of this is a triplication of a large region including the SNCA locus that leads to an early-onset Parkinson’s disease phenotype (Devine et al. 2011). iPSC-derived dopaminergic neurons derived from these patients show molecular phenotypes characteristic of the disease, suggesting that such pathological events can be detected and monitored (Chung et al. 2013). An alternative strategy is to accelerate ageing or disease progression using stressors such as rotenone, MG-132 or concanamycin A (Cooper et al. 2012; Nguyen et al. 2011), or through expression of Progerin, a truncated form of lamin A that is associated with Hutchinson–Gilford progeria syndrome, a premature ageing disorder (Miller et al. 2013). Whilst Progerin expression has been shown to accelerate cellular markers of ageing such as DNA damage and heterochromatic chromatin modifications (Miller et al. 2013), it is still unclear to what extent such treatments fully recapitulate the effects of old age.

Importantly, it is perhaps unsurprising that even with late-onset diseases, there are pathogenic changes occurring at an early point in disease progression that are detectable in iPS models, and can be reverted by pharmacological intervention (reviewed in (Avior et al. 2016)). Arguably, these early changes are more critical in terms of understanding and treating the disease. Studying such effects would facilitate discovery of biomarkers that identify those patients at risk and allow development of strategies to enable early, targeted intervention to prevent the disease. This is particularly important in situations such as neurodegeneration where, by the time patients present with the disease, they often have irreparable damage such as the loss of neurons, and for whom therapeutic intervention at this late stage may not be possible.

### Limitations and developments

**Fig. 1:**
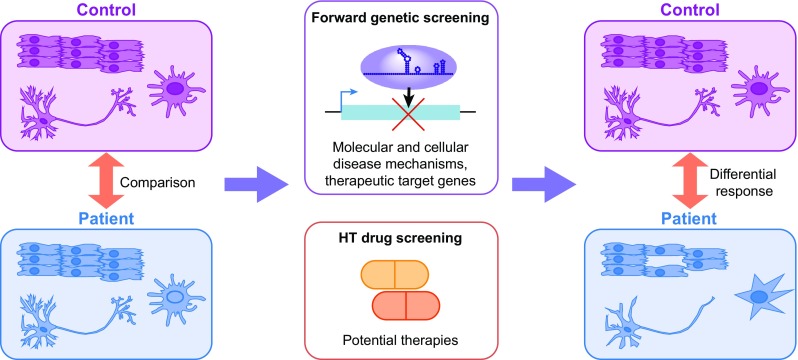
Application of iPS models of disease to high throughput screening. Cells derived from patients with disease and healthy controls can be used to generate disease-relevant cell types, which can be phenotypically compared with each other. Such cells can be generated in sufficient numbers to be able to perform whole genome genetic screens to identify molecular and cellular mechanisms of disease and therapeutic targets, and also for high throughput drug screening to identify compounds that may be able to revert the disease phenotype. Differences between patient-derived and control cells can be used to identify potential therapeutic targets or agents

Whilst the benefits of iPSC technology are undeniable, there are some limitations in their use for modelling of certain disease states. Such in vitro models have immense power in terms of scalability, and being able to apply techniques such as high throughput genetic or pharmacological screening that would not be possible or be technically difficult in an in vivo setting (Fig. 1). However, they are limited in their ability to recapitulate complex tissue architecture both in terms of the complexity of cell types as well as their spatial organisation, making analysis of many physiological or system-level phenotypes challenging. Highly defined co-culture systems can be beneficial in some situations, for instance where the effects are non-cell autonomous, or rely on cell–cell signalling. This has been successfully applied to modelling of the effects of SOD1 mutation in glial cells on motor neuron survival in cells derived from ALS patients (Di Giorgio et al. 2007, 2008).

Exciting developments in terms of three-dimensional culture systems such as intestinal (Dekkers et al. 2013; Schwank et al. 2013) or cerebral (Lancaster et al. 2013) organoids allow analysis of cell–cell interactions in a more complex mixture of cell types with some underlying tissue architecture (Huch and Koo 2015; Lancaster and; Knoblich 2014; Passier et al. 2016). Such systems have been exploited to uncover cellular phenotypes underlying microcephaly (Lancaster et al. 2013) and cystic fibrosis (Dekkers et al. 2013; Schwank et al. 2013), respectively. Although progress is being made in organ reconstruction of structures such as the integumentary system is being made (Takagi et al. 2016), it is unlikely that very complex tissues will be able to be modelled successfully in vitro at least in the near future.

In certain instances, diseases manifest as complex physiological or behavioural phenotypes, which cannot be recapitulated in any in vitro model. An alternative strategy is to use xenograft systems in animal models where the endogenous organ has been genetically ablated (Kobayashi et al. 2010; Lee et al. 2014; Nagashima and Matsunari 2016). These may provide an opportunity to analyse such complex physiological and system-level phenotypes with human patient-derived cells, and potentially provide a source of such organs for transplantation in the future. Whilst such systems may be important for analysis of certain diseases, it is likely that most physiological defects result from inherent underlying molecular or cellular abnormalities. Therefore, cellular phenotypes such as gene expression changes or neuronal electrophysiology will not only help to ascertain the molecular mechanisms underlying complex disease phenotypes such as Alzheimer’s disease but also provide convenient measures for measuring the effects of genetic or pharmacological intervention.

## Genome editing in iPSC disease modelling

### Importance of genome editing

**Fig. 2:**
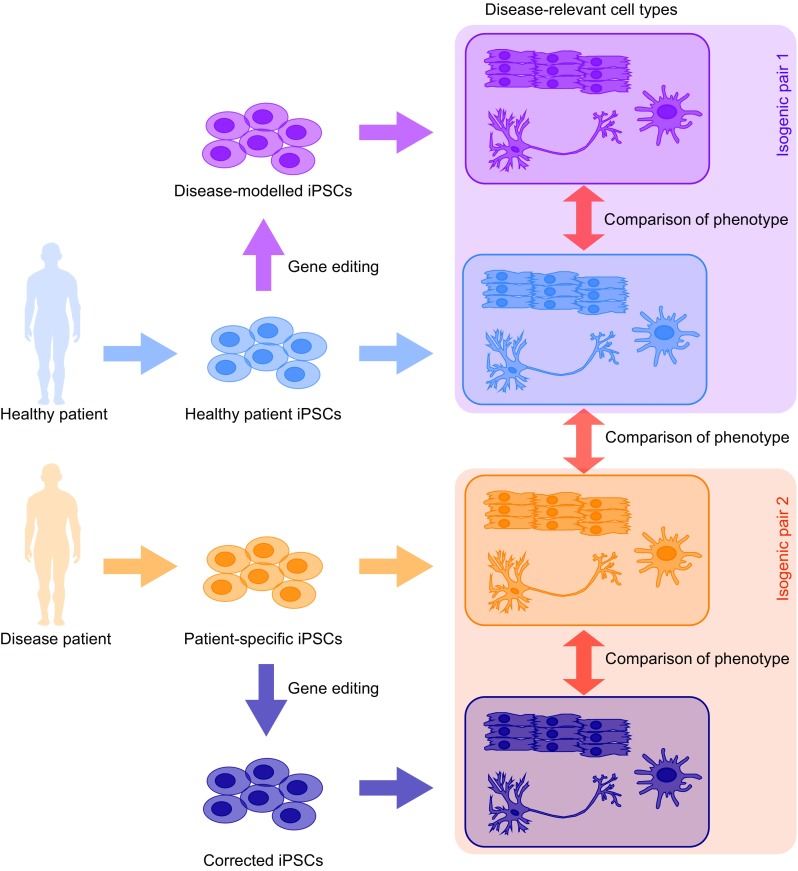
Importance of genome editing in iPS disease modelling. iPSCs can be derived from healthy (*blue*) and disease (*orange*) patients, and after differentiation into an appropriate cell type, comparison of molecular or cellular phenotypes can be made. To minimise variability due to genetic background, genome editing can be used to either correct patient-derived cells (*dark blue*) or to introduce putative causative lesions into cells derived from healthy individuals (*purple*). This leads to isogenic pairs of cell lines (*purple box* or *orange box*) that identify the true impact of the engineered change on the cellular phenotype

Many human genetic diseases by their very nature would only be expected to show subtle effects on cellular behaviour, since those individuals show essentially normal differentiation, development and cellular function and only present symptoms of disease after birth, in old age, or upon exposure to environmental triggers. This alongside an inherent variability in both the iPSC derivation process and differentiation into specific cell types makes it necessary to perform comparisons of many independently derived cell lines from multiple healthy and diseased individuals in order to detect such subtle changes. This can be ameliorated by genetic engineering to introduce or repair putative causative alleles to generate isogenic cell line pairs that have identical genetic backgrounds, and differ in only a single genetic change (Fig. 2). This allows detection of subtle phenotypes that would otherwise be masked by variations in cellular phenotype due to the different genetic backgrounds of the donors.

**Fig. 3:**
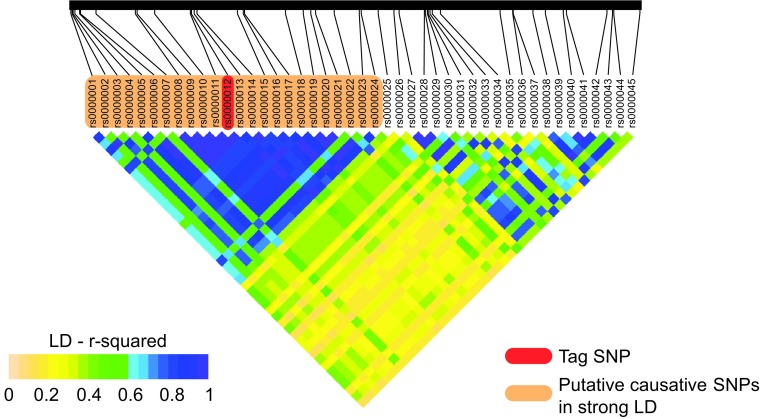
Linkage disequilibrium (LD) makes identification of causative SNPs challenging. In a typical region of the human genome, many SNPs (*orange box*) are in strong LD with the tag SNP (*red*) identified by a GWAS study. Genome editing can be used to identify the causative lesion from within this LD block. LD is measured as R-squared values between pairs of SNPs, and indicated on the heatmap

Importantly, such experiments also allow the identification of the causative lesion that defines the cellular phenotype and results in the disease (Nishizaki and Boyle 2016). This is not possible from comparisons of iPSC lines derived from patients with the disease and healthy controls, due to the inheritance patterns of linked SNPs. Often several SNPs are in linkage disequilibrium (LD) with each other, and as such are always inherited together (Weiss and Clark 2002) (Fig. 3). Deciphering which of these are causative is therefore a challenge, and although it is possible to infer some information from their position relative to known important genomic features (e.g. protein coding sequence, DNAse hypersensitive sites, etc.), experimentally identifying the important genetic aberration is still not trivial. Genome editing allows the attractive possibility of either repairing the putative causative lesions in patient-derived cells, or introducing them in cells derived from healthy individuals (Fig. 2), to unambiguously identify the mutations involved in the disease phenotype.

Genome editing has been used in many examples (Table 1) to create isogenic pairs of cell lines, and this has been successful in both validating the causative lesion and allowing greater sensitivity for phenotypic detection. The importance of such studies is undeniable, and the usage of such isogenic lines in iPS disease modelling will undoubtedly increase in the coming years.

**Table 1:** iPSC disease models employing isogenic control lines generated by CRISPR/Cas9. The table lists a number of diseases, the mutation that was reverted or introduced, the differentiated cell type analysed and the molecular or cellular phenotypes observed

Disease	Editing	Cell type	Phenotype	References
Neurological disorders				
Fragile X syndrome	Removal of triplet repeat (FMR1)	NPC neurons	DNA methylation, gene expression changes	(Boland et al. 2017; Xie et al. 2016)
Tetrahydrobiopterin metabolism disorder, Parkinson’s disease	Correction of dopamine synthesis (PHPS, DHPR)	DA neurons	Metabolic (dopamine, BH4), protein expression changes	(Ishikawa et al. 2016)
Parkinson’s disease (PD)	Correction of coding point mutations (SNCA)	Cortical neurons	Accumulation of ER-associated degradation substrates	(Chung et al. 2013; Soldner et al. 2011)
	Correction of coding mutation (LRRK2)	Dopaminergic neurons	Gene expression changes	(Reinhardt et al. 2013)
Alzheimer’s disease (AD)	Point mutations (APP, PSEN1)	Cortical neurons	Protein (Aβ) secretion	(Paquet et al. 2016)
Hereditary motor and sensory neuropathy with proximal dominant involvement (HMSN-P)	Correction of point mutations	Spinal motor neurons	Proteasome impairment	(Murakami et al. 2017)
Frontotemporal lobar degeneration tauopathy (FTLD-Tau)	Correction of intronic or exonic point mutation (MAPT)	Neurons	Accumulation and release of misfolded tau, cell death, electrical stimulation of calcium transients	(Imamura et al. 2016)
Huntington disease (HD)	Correction of expanded CAG repeat (HTT)	Forebrain neurons	Neural rosette formation, mitochondrial respiration, gene expression changes	(Xu et al. 2017)
Immunodeficiency, centromeric instability, facial abnormalities (ICF) syndrome	Knockout (DNMT3B)	iPSC	Gene expression, DNA methylation changes	(Horii et al. 2013)
Other diseases				
Chronic granulomatous disease (CGD)	Repair of intronic point mutation, exon 5 replacement (CYBB)	Phagocytes	Oxidative burst function (ROS release)	(Flynn et al. 2015; Sweeney et al. 2017)
Duchenne muscular dystrophy (DMD)	Exon skipping, frameshift, exon knock-in, deletion (Dystrophin)	Skeletal muscle	Membrane integrity, protein expression, electrical contraction (Ca overflow) changes	(Li et al. 2015; Young et al. 2016)
Barth syndrome	Knockout (TAZ)	Cardiomyocytes	Metabolic and structural changes in muscle, myocardial contraction defects	(Wang et al. 2014a, b)
Monocytic and dendritic cell immunodeficiency	Knockout (IRF8)	Dendritic cells	Differentiation, cytokine release, migration, antigen presentation	(Sontag et al. 2017)
β-thalassemia	Correction of coding mutation (HBB)	Erythroblast	Gene expression changes, hematopoietic differentiation, reactive oxygen species production	(Niu et al. 2016; Song et al. 2015)
Hemophilia A	Reversion of chromosomal inversion	Endothelin cells	Restoration of factor VIII expression, rescue of lethality in mouse xenograft model	(Park et al. 2015)
Severe combined immunodeficiency (SCID)	Repair of loss of function mutation (JAK3)	T-cells	Restoration of correct T-cell development	(Chang et al. 2015)
Sickle Cell Anaemia	Reversion of point mutation (HBB)	Erythrocytes	HBB expression	(Huang et al. 2015)

### CRISPR genome editing technology

Recent improvements in genome editing have vastly increased our ability to introduce such delicate defined mutations within the genomes of human cells. These are based on designer site-specific nucleases that introduce a double-strand break (DSB) at a desired site in the genome, which is then repaired by the cell, and can be utilised to introduce a variety of different genetic changes at this site. Most recent work focuses on the clustered, regularly interspaced short palindromic repeats (CRISPR) system and the use of the RNA-guided CRISPR-associated 9 (Cas9) endonuclease (Cho et al. 2013; Cong et al. 2013; Jinek et al. 2012, 2013; Mali et al. 2013), predominantly due to the simplicity by which this can be reprogrammed to bind to millions of sites within the genome. This system relies on a short guide RNA molecule to direct its specificity, through base pairing of its first 20 nt with the corresponding DNA sequence in the genome (Jinek et al. 2013). The only limitation to the targeting is a requirement for a protospacer adjacent motif (PAM) sequence adjacent to the target site, which is not present in the guide RNA, but is recognised by the Cas9 protein. In the case of the most widely used *Streptococcus pyogenes* Cas9 protein, this is NGG, which in a genome with an even base distribution and composition should occur every 8 bp. However, should this be a limitation, orthologues of Cas9 in other species have been discovered with alternative PAM requirements (Hou et al. 2013; Ran et al. 2015; Zetsche et al. 2015) and recently, protein engineering has been used to alter the PAM sequences of several Cas9 nucleases (Kleinstiver et al. 2015a, b) (as discussed elsewhere in this issue).

Introduction of a DSB into the genome allows many different types of genetic manipulation. Usually, this DNA damage is repaired by one of two major pathways, non-homologous end joining (NHEJ) that performs non-templated ligation of the free DNA ends, and homology directed repair (HDR) that utilises homologous DNA to direct a precise repair (Bibikova et al. 2003; Shrivastav et al. 2008). Both of these can be exploited for making site-specific genetic mutations. Repair through NHEJ or related pathways such as microhomology mediated end joining (MMEJ) can result in insertions or deletions (indels) of several nucleotides at the DSB that can be used for instance to introduce a frameshift in protein coding sequence, resulting in a null allele. If two DSBs are made simultaneously, the NHEJ machinery can also ligate the wrong termini together, resulting in deletions, inversions or translocations (Torres et al. 2014; Xiao et al. 2013; Park et al. 2016). In the context of more defined changes to the DNA such as introduction of SNPs, HDR pathways can be utilised by providing an excess of a desired sequence, resulting in this being used in preference to the sister chromatid as the template for repair. Introduction of mutations can be highly efficient, especially for NHEJ-based pathways, which generally predominate in most cells including human iPSCs, and can approach 80–90% in the best examples.

This efficiency, coupled with the simplicity of constructing guide RNAs from synthetic oligonucleotides, which are amenable to being produced in both multi-well plates or in larger pools of tens of thousands, has allowed both arrayed (Hultquist et al. 2016) and pooled screening strategies (Gilbert et al. 2014; Koike-Yusa et al. 2014; Konermann et al. 2015; Shalem et al. 2014; Wang et al. 2014a, b) to be conceived. This provides the exciting prospect of forward genetic screening in human patient-derived cell lines to identify potential therapeutic targets and to better understand molecular and genetic basis of disease (Fig. 1).

Genome editing technologies also have the potential in the future to be utilised as a therapeutic agent in their own right, and to repair the genetic mutations contributing to disease (Cox et al. 2015). Such reagents could not only be applied in an in vivo context, but also to repair causative lesions in patient-derived iPSCs that could subsequently be used to generate specific cell types to use as cellular therapies. Such strategies have shown significant promise in some cases, for example in the treatment of a chemically induced primate model of Parkinson’s disease through injection of autologous iPSC-derived dopaminergic neurons (Emborg et al. 2013; Hallett et al. 2015). Therapies for HIV (Tebas et al. 2014) and cancer (Fesnak et al. 2016) involving genome editing are already in clinical trials, and the next few years will likely herald exciting developments in this area of somatic gene therapy.

## Strategies for genome editing in iPS models of disease

There are two main strategies for using genome editing techniques in iPSC models of disease. The first involves repairing a pre-existing, presumed causative allele from an iPSC line derived from a patient with the disease (Fig. 2, isogenic pair 1). This establishes whether this particular genetic change contributes to the disease phenotype, but does not provide any information about whether it is sufficient to cause disease. It also has the substantial benefit that the patient-derived cell line would be expected to express whatever cellular or molecular phenotype that is causing the disease, and therefore reversion of this phenotype in the edited line can be used as a readout. The second strategy involves taking an iPSC line from a healthy patient, and introducing a putatively important lesion (Fig. 2, isogenic pair 2). This is perhaps a more stringent assay, since it establishes whether this single genetic change is sufficient to cause the disease phenotype, since it removes it from the genetic background of the diseased individual. However, if no effect is seen on the molecular or cellular phenotype of interest, it is not possible to infer whether this allele contributes to the disease. Equally, the effect of genetic background can be investigated in this manner by introducing putative causative lesions into a panel of “WT” iPSCs established from healthy donors from diverse genetic ancestries.

These strategies are clearly complementary to each other, and can provide information on potential epistatic interactions with other alleles present in specific genetic backgrounds. It is also worth considering that modification of a “WT” cell line is often simpler technically, since both the genome editing and subsequent downstream differentiation and analysis can be optimised for a particular cell type. Additionally, the “WT” cells can be thoroughly characterised beforehand, and different genetically edited lines involved in the same disease can be directly compared. In the case of patient-derived cells, each cell line will behave somewhat differently, and therefore it is often more difficult to perform such manipulations, especially at higher throughput.

When designing such an experiment, it is also important to consider the inherent variability between patients as well as in the processes of both reprogramming of somatic cells to iPSCs and differentiation into particular cell types. It has been suggested that at least part of this heterogeneity results from an “epigenetic memory” of the DNA methylation signature of the cell type from which the iPSCs were derived (Kim et al. 2010), which may impact upon the phenotypes observed, or the ability to differentiate into particular lineages (Bar-Nur et al. 2011). Reassuringly however, recent studies looking at the origin of heterogeneity within 25 iPSC lines have demonstrated that, at least at the transcriptional level, the majority of variation is due to genetic background as opposed to any epigenetic contribution (Rouhani et al. 2014). In order to account for such inherent variability, it is necessary to analyse multiple (typically at least three) patients, each with independent iPSC derivations, clonally derived lines and differentiation experiment, which rapidly increases the number of samples that need to be analysed (Fig. 2). The number of each that are required depends on multiple factors including the magnitude of the phenotype and the degree of variability within a particular differentiation protocol, but it is unwise to rely at any stage on the results from a single experiment.

Whilst genome editing can be very effectively used to reduce the variability between patients, the process of genome editing itself can introduce artefacts from both off-target mutagenesis (Cradick et al. 2013; Fu et al. 2013; Veres et al. 2014) and clonal variability within a particular iPSC line. One method for controlling the variability introduced by the genome editing process is by re-introducing the disease mutation in the genetically corrected patient line. Similarly, introducing mutations into a consistent “WT” line can reduce the variability between patients and during iPSC derivation. The experimental strategy will depend on the specific question that is being addressed, but these criteria should be taken into account in order to maximise sensitivity for phenotypic changes, and minimise workload.

## Genome editing methods

Numerous strategies exist for genome editing using CRISPR, predominantly differing in the method of delivery of the Cas9 and guide RNA components. Inducible Cas9 transgenes (Gonzalez et al. 2014), DNA plasmids (Ding et al. 2013a, b; Kwart et al. 2017; Merkert et al. 2014; Miyaoka et al. 2016, 2014; Yang et al. 2013) or Cas9 ribonucleoprotein (RNP) complexes (Kim et al. 2014; Liang et al. 2015; Lin et al. 2014; Richardson et al. 2016) have all been successful in introducing targeted mutations in hiPS cells (reviewed in more detail in Merkert and Martin 2016; Santos et al. 2016). The choice of system depends on multiple factors including the importance of off-targeting, the type of repair necessary (single nucleotide changes or indel mutations) and prior knowledge of optimal delivery methods into a particular cell line. However, our preference is for delivery of RNPs composed of recombinant, bacterially expressed Cas9 protein and synthetic RNA oligos corresponding to the CRISPR RNA (crRNA) and trans-activating CRISPR RNA (tracrRNA) components of the system. This has many advantages over other systems since the RNP complex is immediately active, when the concentration of any donor HDR template is the highest and is rapidly degraded over a period of around 12 h (Kim et al. 2014), reducing the potential for off-target mutagenesis and re-targeting after successful HDR. The lack of DNA plasmids also eliminates any chances of non-specific integration of DNA vectors into the genome.

Another important consideration is to minimise off-target mutagenesis, the extent of which is still debatable in the field, and likely depends on the exact system used to introduce the CRISPR reagents (Cradick et al. 2013; Fu et al. 2013; Veres et al. 2014). What is clear is that some mismatches between the guide RNA and target DNA can be tolerated, and the degree to which they impact on endonuclease activity depends on their position within the sequence, with those nucleotides closer to the PAM sequence playing a more critical role in target recognition (Hsu et al. 2013). Careful design of crRNA target sites to avoid off-targets of less than 3 mismatches can be readily achieved using a variety of online tools and will certainly minimise any potential problems. Methods for improving specificity have been developed using either pairs of Cas9 enzymes each of which is unable to generate a DSB alone (Guilinger et al. 2014; Ran et al. 2013; Tsai et al. 2014), truncated guide RNAs (Fu et al. 2014) or protein engineering of Cas9 to improve specificity (Kleinstiver et al. 2016; Slaymaker et al. 2016). Some of these strategies including the double nickase approach (Ran et al. 2013) have been successfully used in hiPS cells (Eggenschwiler et al. 2016; Wu et al. 2016b), although these systems will never remove the potential for off-target mutations completely. Therefore at least in the case of disease models, where a small number of lines are produced, it is also possible to sequence the putative off-target sites (or even the whole genome), generate cell lines with independent guides each of which would have a different set of off-targets, or perform another round of genome engineering to repair the introduced genetic change.

Any such manipulations generate a mixed population of cells with different genotypes, and a large proportion of the workload is in clonal growth of cells and genotyping them. Thus, strategies for rapid, scalable and efficient genotyping are paramount to the success of any genome editing experiment. Numerous techniques can be employed, either at the DNA, RNA, protein or functional level. Often selection strategies begin with PCR amplification of regions around the sgRNA target site, and subsequent analysis by restriction enzyme polymorphisms, digital PCR (Mock et al. 2016) and Sanger (Brinkman et al. 2014) or high throughput sequencing (Bell et al. 2014). However, the choice of strategy will largely depend on the efficiency of the process, and the class of mutant introduced.

### Gene knockouts

**Fig. 4:**
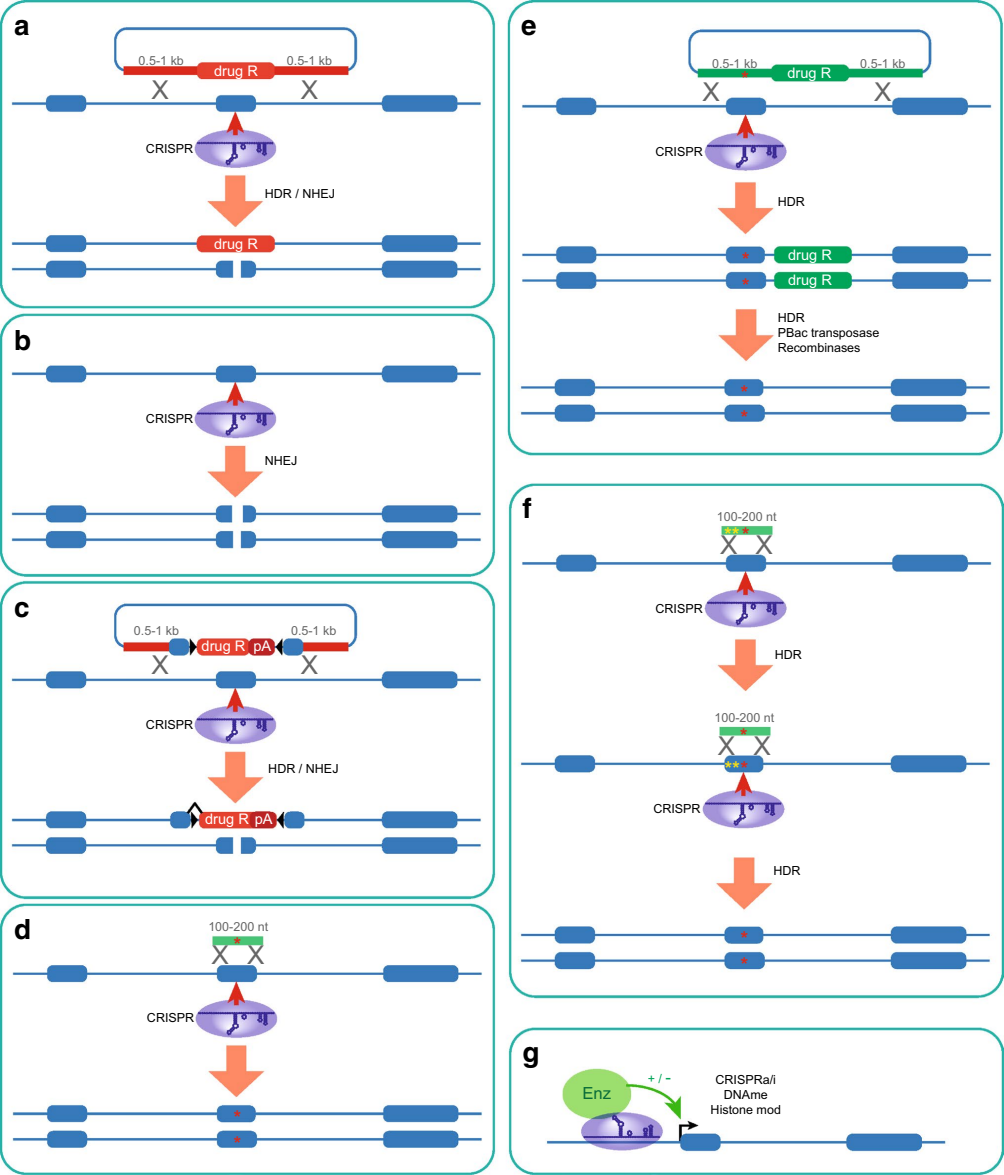
Strategies for genome editing using CRISPR/Cas9. **a** Gene knockout—CRISPR-enhanced HDR can be employed to replace a critical exon with a selectable drug resistance cassette (drug R), on one allele, relying on NHEJ-dependent indels to disrupt the other allele. **b** Gene knockout—A CRISPR-induced DSB can be used to efficiently introduce indels on both alleles. **c** Conditional knockout by inversion (COIN)—CRISPR-enhanced HDR can be used to introduce a Cre-recombinase invertible cassette, flanked by loxP sites (*black triangles*) into an artificial intron. This contains a splice acceptor site followed by a transcriptional termination signal (pA), so in one orientation it causes premature termination and mutation of the gene. In the opposite orientation, splicing occurs around the cassette, allowing the normal gene product to be produced from this allele. The second allele is disrupted by NHEJ-induced indels as in (**a). d** SNP introduction—A CRISPR-induced DSB is used to enhance HDR with a 100–200 nt ssDNA oligonucleotide repair template (*green*) to introduce small defined changes. **e** Scarless SNP introduction—A selectable marker cassette (drug R, *green*) is introduced into an intron or non-functional region along with the SNP of interest, and subsequently removed by a further round of HDR, or the piggyBac transposase. **f** Scarless SNP introduction—A SNP of interest is introduced as in D along with second site mutations necessary to prevent re-cleavage by the Cas9 enzyme. A subsequent second round of editing in a similar manner corrects the secondary mutations to leave only the SNP of interest. **g** Epigenetic editing—Catalytically dead Cas9 protein is used to recruit a variety of enzymatic activities (Enz, *green circle*) to specific sites, leading to transcriptional modulation (both positively and negatively), DNA or histone modifications such as DNA methylation, histone acetylation, methylation or phosphorylation, or cytosine deamination

Different classes of allele vary in terms of the ease by which they can be generated, largely due to the bias of repair pathways towards NHEJ, and the resulting indel mutations. Gene knockouts are perhaps the simplest to produce, since such indel mutations can be used to introduce frameshifts into protein coding sequence (Fig. 4b), making the efficiency of mutagenesis very high. It is important to carefully consider the gene structure when designing such a strategy, since alternative promoters, alternative splicing and alternative polyadenylation signals are all critical factors to consider.

It is important that frameshifts are not made too early in the protein coding sequence, since if the open reading frame (ORF) is too short, reinitiation at a downstream start codon can occur (Zimmer et al. 1994). The efficiency of such reinitiation is dependent on the length of the upstream ORF, and is highly inefficient if this is greater than 30–40 amino acids (Luukkonen et al. 1995). Equally, frameshifts should not be introduced too late in the coding sequence. Normally premature stop codons are recognised by the cell and trigger the process of nonsense mediated decay (NMD), which prevents expression of the entire protein through mRNA degradation (Hug et al. 2016). However, if the premature stop codon is present in the final exon, this process does not occur, and a slightly truncated peptide will be produced, likely with at least partial functionality (Hug et al. 2016). In order to obtain a complete knockout allele, it is therefore optimal to target a constitutive exon at least 30–40 amino acids into the protein coding sequence that is not in the final exon. Even in this situation, a complete knockout cannot be guaranteed, since NMD does not always act efficiently, and aberrant splicing can occur to reconstitute at least part of the protein function. Thus, analysis of protein expression is an important aspect to consider with such mutations.

Many other strategies exist, such as removal of constitutive exons through NHEJ-mediated deletions with pairs of guide RNAs flanking these exons (Liu et al. 2016a, b), or using a homology construct that guides precise exon deletion of one allele (Fig. 4a), coupled with indel mutations on the other. These systems can also be used to make larger deletions to recapitulate for instance copy number variants (CNVs), which could be informative in certain cases. In all cases, it is also important to consider whether the knockout of the gene will be lethal at a cellular level, in which case conditional knockout by for instance flanking constitutive exons with recombinase sites, or conditional by inversion (COIN) or FLIP strategies (Economides et al. 2013; Andersson-Rolf et al. 2017) may be beneficial (Fig. 4c). Inducible CRISPR systems can also be employed to restrict mutations to particular points in time (Bertero et al. 2016), and may be helpful in certain situations.

### Single nucleotide polymorphisms

In many cases, genetic changes identified from familial inheritance studies or GWAS are single nucleotide polymorphisms (SNPs). Such mutations often do not result in complete loss of protein function, and frequently cause missense mutations in protein coding sequence, or changes at non-coding regulatory sites that may affect for instance transcription factor binding.

In order to introduce such delicate changes to the genome, it is necessary to employ the HDR pathway of DNA repair, making this process less efficient in general than simple gene knockouts (Fig. 4d). Templates for DNA repair can be supplied as either double-stranded DNA plasmids with approximately 500–1000 nt of homology either side of the introduced mutation, or more typically as chemically synthesised short single-stranded DNA oligonucleotides (ssODN) of 100–200 nt in length. The latter are simple to design and synthesise and have a comparable efficiency of HDR to longer dsDNA fragments due to the higher recombinogenic activity of ssDNA (Chen et al. 2015). Although there is substantial variability in the absolute efficiencies reported in the literature, we find similarly to others that a combination of chemically synthesised crRNA and tracrRNA, recombinant Cas9 protein and approximately 100 nt ssODN highly effective for introduction of SNPs (Kim et al. 2014; Liang et al. 2015; Lin et al. 2014; Niu et al. 2016; Richardson et al. 2016; Song et al. 2015). This obviates the need for any cloning or DNA manipulation, and all components can be purchased from commercial vendors. However, longer homology constructs have also been effectively used to introduce point mutations in other published reports (Wang et al. 2017; Yusa 2013).

In all cases, it is critical to ensure that upon correct HDR, the sgRNA is unable to guide further DSB events on the newly modified allele, since these could result in further undesirable mutagenesis through NHEJ-mediated indels. This ideally requires that the sgRNA is chosen to span the SNP of interest. Since the guide RNA can tolerate up to three mismatches whilst still retaining the ability to direct the Cas9 endonuclease to this site, it is often necessary or advisable to introduce additional base changes outside the SNP of interest to ensure this is the case. For SNPs within protein coding genes, this can be achieved by introducing silent mutations into the DNA whilst maintaining its protein coding capacity. Even in this case, there may still be a role of such synonymous mutations in protein translation efficiency (Quax et al. 2015), exonic transcription factor binding (Stergachis et al. 2013) or other as yet unknown functions. However, for those changes outside of protein coding sequence, it is usually impossible to predict the effects of such secondary mutations. This makes it necessary to either perform completely scarless mutagenesis or alternatively to create two independent alleles with different secondary mutations, which can be used to control for the effects of these mutations.

Scarless mutagenesis is possible in some cases where the SNP happens to fall within the PAM sequence, or within a few nucleotides of the 3′ end of the guide RNA, since even single-point mutations at these sites can prevent re-cleavage. However, this is not the case in many instances, and certain nucleotides are therefore inaccessible to such manipulations at least with *Streptococcus pyogenes* Cas9. As mentioned above, orthologues from other species (Hou et al. 2013; Ran et al. 2015; Zetsche et al. 2015) or engineered variants (Kleinstiver et al. 2015a, b) are already increasing the range of potential targets, and it is likely that in the future, most SNPs will be amenable to manipulation in this manner. Another strategy is to use a larger dsDNA donor to introduce a selectable marker cassette into a non-functional region neighbouring the SNP of interest (Fig. 4e) (Wang et al. 2017; Yusa 2013). This cassette can subsequently be removed either by site-specific recombinases, or scarlessly by a second round of homologous recombination or the piggyBac transposase (Wang et al. 2017; Yusa 2013). However, in the latter case, there are sequence requirements on where the piggyBac sequences can be integrated that may limit the effectiveness of this strategy (Yusa 2013). A third strategy involves a two-step genome editing strategy whereby in the first step the desired mutation is introduced alongside secondary mutations to prevent recutting, and subsequently, the secondary mutations are removed by a redesigned guide (or alternative Cas9 enzyme) and homology template (Fig. 4f) (Paquet et al. 2016; Kwart et al. 2017). However, these latter strategies involve considerably more complex processes, and at least two stages of clonal selection, increasing the time and cost of producing each mutation.

An additional strategy which has recently been developed is the use of Cas9 to recruit cytosine deaminase enzymes usually involved in somatic hypermutation in immune cells (such as the Apobec and AID enzymes) to edit the sequence of the genome without inducing a DSB (Hess et al. 2016; Komor et al. 2016; Nishida et al. 2016) (Fig. 4g). Whilst this could be informative in terms of screening and for certain specific mutations, its general application in disease modelling is somewhat limited, since only transitions from cytosine to thymine are possible.

### Epigenome editing

In addition to genetic manipulations, such as gene knockouts or single nucleotide changes, a growing number of diseases including cancer (Salarinia et al. 2016) and neurodegenerative (Gos 2013; Jakovcevski and Akbarian 2012; Landgrave-Gomez et al. 2015) diseases can be driven by changes in the epigenome of the cell. This can be both in terms of transcriptional levels, or for instance changes in DNA methylation patterns that may have more subtle effects, e.g. to alter how the cell responds to external signals. The impact and importance of such changes can be assessed by using nuclease-deficient forms of Cas9 fused to specific chromatin or DNA modifying factors (Fig. 4g). Two point mutations in the Cas9 protein (D10A, H840A) render it catalytically inactive, whilst retaining its ability to bind to specific DNA sequences. Fusion of domains to the Cas9 protein or binding to modified guide RNA scaffolds then allow recruitment of specific enzymatic activities to desired sites in the DNA (Dominguez et al. 2016; Konermann et al. 2013) (Fig. 4g). This has been used extensively to manipulate gene expression both positively using transcriptional activation domains such as VP16, Rta, p65 or HSF1 and negatively by fusion to KRAB, and genome-wide screens using these reagents have been successfully implemented (Gilbert et al. 2014; Konermann et al. 2015). Such systems have been used to for instance manipulate alpha-synuclein levels both positively and negatively in neurons derived from patients with a triplication of the SNCA locus, allowing manipulation of SNCA levels in this disease model (Heman-Ackah et al. 2016). Equally importantly, a variety of chromatin modifying activities can also be recruited, including the p300 histone acetyltransferase (Hilton et al. 2015) or LSD1 histone demethylase (Kearns et al. 2015) to activate or inactivate enhancers, the Dnmt3 DNA methyltransferases (Amabile et al. 2016; Liu et al. 2016a, b; Vojta et al. 2016) or TET1 demethylase (Choudhury et al. 2016; Liu et al. 2016a, b; Xu et al. 2016) to add or remove DNA methylation marks, or other chromatin modifying enzymes such as G9a or SUV39H1 (Snowden et al. 2002). These reagents will be important in establishing the role and importance of such epigenetic changes in disease progression either by introducing them into healthy cells, or reverting effects observed in disease models.

## Conclusions

The recent advances in DNA sequencing technologies have led to an ever increasing number of human genetic studies that have identified numerous candidate loci that are correlated with many diseases. There is therefore a pressing need for simple, robust models of disease that can be applied to understand the functionality of such genetic lesions. It is clear that the intersection of iPSC and genome editing technologies will provide powerful tools to study such diseases in a human cellular system. The use of a human system has many advantages, especially in terms of studying the majority of disease-associated mutations that do not reside within protein coding genes, and for which conservation is not sufficient to allow direct comparisons to be made in other organisms. The ability to be able to derive cells from multiple patient genotypes and the ability to obtain such cell lines from repositories around the world will provide invaluable opportunities to investigate the link between genotype and phenotype. Genome editing will form an essential component of such studies, to revert or introduce desired genetic mutations to understand their function by comparison with a fully isogenic background (Fig. 2). iPSC technologies are also amenable to higher throughput studies of multiple SNPs, identification of the causative lesions, loss- and gain-of-function genetic screens to understand disease mechanisms and identify drug targets and high throughput drug screening, which are likely to be highly informative in the coming years (Fig. 1).

One of the powers of iPSCs is that all of these strategies can also be applied to multiple different cell types, although it remains to be seen to what extent the results obtained in such isolated cell populations recapitulate the effects seen in complex human tissues. As differentiation protocols and three-dimensional cell culture techniques evolve, this can only improve our ability to use such systems to model many aspects of human disease. However, such cellular systems will always be limited in terms of assaying more complex system-level physiological and behavioural phenotypes, although xenograft systems may provide one means of achieving this in an in vivo context. One of the most important aspects of employing iPSC models of human disease is therefore in cellular and molecular phenotyping. Whilst for many diseases, such phenotypes will be known and predictable, for others this will require correlation of the normally complex physiological phenotypes seen in disease to the underlying molecular and cellular defects. Whilst challenging, this will also provide invaluable information about the pathogenic mechanisms leading to such diseases and novel avenues for therapeutic intervention.

The synergy between iPSC and genome editing technologies will no doubt provide many insights into disease mechanisms and therapeutic targets, and enable characterisation and prioritisation of genetic aberrations that cause particular diseases. This information coupled with the applications of iPSC-derived cell types for cellular therapies and CRISPR-based reagents for in vivo therapeutics offer exciting possibilities for personalised genetic medicines in the not-too-distant future.
